# Correspondence

**Published:** 1870-07

**Authors:** F. K. Bailey

**Affiliations:** Knoxville, Tenn.


### Correspondence.

---

Knoxville, Tenn., *May 18th, 1870.*

Prof. Davis,—The Spring has been unusually cool and wet. Until April 20th fires were necessary, and snow was seen upon the Unaka Mountains.

As a result, catarrhal affections, to which allusion was made in the March number of the *Examiner*, continued to prevail, and, in some instances, became pneumonia of a decidedly typhoid type. Many cases were continued or remittant and, disposed to resist treatment. The prominent characteristics were constipation, urine scanty, red, and acid, breathing oppressed and rapid, with a tendency to coldness of the extremities.

The blood seemed poisoned by effete matter, which the emunctories failed to eliminate.

There was copious secretion from the bronchial mucus membrane, which, in children too young to throw it from the mouth, caused much gastric disturbance; the torpidity of the alimentary canal rendered cathartics a daily necessity, and generally mercurials had the best effect—frequently nothing else would answer the indications.

After the disease, yielded by resolution, or expended itself by critical terminations, sul. quinin, and other tonics were clearly called for. In some cases, there were tertian exacerbations, and partial remissions of excitement, when quinin, in decided doses, had the effect to suspend somewhat the morbific influence and accelerate a cure which nature was striving to effect at her more deliberate rate.

In many localities in E. Tenn., pneumonia has prevailed extensively and proved fatal.

There is a sthenic condition in about every case of disease met here, and a decided antiphlogistic course of treatment is demanded. Venesection is rarely employed by the old resident physicians; but calomel and other reducing agents are freely administered. To one who, for nearly thirty years, had practiced in the North-West, or *interior* more properly speaking, and met with nothing but the results of malaria, it is an agreeable change to be located where inflammatory diseases are common.

Roseola prevailed epidemically in April, whole families becoming effected simultaneously. The rash came out very thickly, and continued for a week, but receding and recurring at short intervals. Saline cathartics, with general treatment, called for by each case, soon restored the health.

During the cold and damp weather of Winter and Spring, dysmenorrhea is very common here, and I have found iodide and bromide of potassium very effectual. The former particularly if there was a rheumatic diathesis. Amenorrhea, for similar reasons, is quite often met with, and bromide potassium, with the pil. rhei. comp. and ammoniated tincture of guaiac will generally cause a return of the function. I find that hot whiskey toddy is very commonly used as a domestic remedy in painful menstruation, and some late writer has alluded to the effects of alcohol in that affection, in a favorable manner.

While writing this, I was invited by a medical friend to call with him to see a case of temporary insanity.

The patient is an unmixed African, about thirty years old, strong animal development, low retreating forehead, and lips nearly an inch in thickness. Since Saturday, the 14th inst., he has been wild at times, and to-day became completely frienzied. He complains of his head as if it would burst. But little can be learned of his history, except that during the war he received an injury upon the top of his head.

My friend had given chloral hydrate, in ten grain doses every two hours; and when we called he had taken two doses. He was quiet, with a cool moist skin, pulse not accelerated, but wakeful. The Doctor also gave calomel, in four grain doses, with a-quarter grain of tartar emetic, every two hours.

20th. The patient remained quiet during the night of the 18th, but yesterday, was as wild as ever, and to-day is perambulating the streets with his hand upon his head, complaining of pain. Further inquiry has elicited the fact that during the war he was present at some religious meeting, where he became very much excited, and, that while in this mental condition, he received, by accident or otherwise, a blow upon the head as above stated.

The memory of those circumstances, under which the injury was received, seems still vivid, and intensified by excitement.

His minister states that, of late he has become so much excited upon religious matters, while at church, as to be an object of fear.

He talks incoherently of the circumstances under which the injury was received, and associates with them his religious feelings.

There must be some local cerebral affection, which may, in time, resume its latent state, or disolve itself into a form sooner or later to become fatal. I may report further of this case.

F. K. BAILEY, M.D.

---

MECHANICAL SUPPORT DURING LABOR.—An English obstetrician has contrived an appliance for facilitating labor; which he describes in the *Lancet* as an "*obstetric support*," and which, according to his account, expedites the work of nature in a remarkable manner. It encircles and supports the abdominal muscles, ensures the due contraction of the uterus, compresses the placental vessels and prevents hemorrhage, rectifies dependence of the uterus, and supports the back. What with this support without, the forceps within, and chloroform in the brain, an accouchement is likely to become mere baby-play.—*Pacific Medical and Surgical Journal.*

